# Impact of appropriate empirical antibiotic treatment on recurrence and mortality in patients with bacteraemia: a population-based cohort study

**DOI:** 10.1186/s12879-017-2233-z

**Published:** 2017-02-06

**Authors:** Kim O. Gradel, Ulrich S. Jensen, Henrik C. Schønheyder, Christian Østergaard, Jenny D. Knudsen, Sonja Wehberg, Mette Søgaard, for the Danish Collaborative Bacteraemia Network (DACOBAN), Christian Østergaard, Jenny Dahl Knudsen, Ulrich S. Jensen, Magnus Arpi, Mette Pinholt, Sara Thønnings, Henrik C. Schønheyder, Mette Søgaard, Kristoffer Koch, Jesper Smit, Kim O. Gradel

**Affiliations:** 1 Center for Clinical Epidemiology, South, OUH Odense University Hospital, Kløvervænget 30, Entrance 216, DK-5000 Odense C, Denmark; 2 Research Unit of Clinical Epidemiology, Institute of Clinical Research, University of Southern Denmark, Odense, Denmark; 3 Department of Clinical Microbiology, Slagelse Hospital, Slagelse, Denmark; 4 Department of Clinical Microbiology, Aalborg University Hospital, Aalborg, Denmark; 5 Department of Clinical Medicine, Aalborg University, Aalborg, Denmark; 6 Department of Clinical Microbiology, Copenhagen University Hospital, Hvidovre, Denmark; 7 Department of Clinical Epidemiology, Institute of Clinical Medicine, Aarhus University Hospital, Aarhus, Denmark

**Keywords:** Bacteraemia, Recurrence, Mortality, Population-based, Epidemiology

## Abstract

**Background:**

Data on the impact of empirical antibiotic treatment (EAT) on patient outcome in a population-based setting are sparse. We assessed the association between EAT and the risk of recurrence within one year, short-term- (2–30 days) and long-term (31–365 days) mortality in a Danish cohort of bacteraemia patients.

**Methods:**

A cohort study including all patients hospitalized with incident bacteraemia during 2007–2008 in the Copenhagen City and County areas and the North Denmark Region. EAT was defined as the antibiotic treatment given at the 1^st^ notification of a positive blood culture. The definition of recurrence took account of pathogen species, site of infection, and time frame and was not restricted to homologous pathogens. The vital status was determined through the civil registration system. Association estimates between EAT and the outcomes were estimated by Cox and logistic regression models.

**Results:**

In 6483 eligible patients, 712 (11%) had a recurrent episode. A total of 3778 (58%) patients received appropriate EAT, 1290 (20%) received inappropriate EAT, while EAT status was unrecorded for 1415 (22%) patients. The 2–30 day mortality was 15.1%, 17.4% and 19.2% in patients receiving appropriate EAT, inappropriate EAT, and unknown EAT, respectively. Among patients alive on day 30, the 31–365 day mortality was 22.3% in patients given appropriate EAT compared to 30.7% in those given inappropriate EAT. Inappropriate EAT was independently associated with recurrence (HR 1.25; 95% CI = 1.03–1.52) and long-term mortality (OR 1.35; 95% CI = 1.10–1.60), but not with short-term mortality (OR 0.85; 95% CI = 0.70–1.02) after bacteraemia.

**Conclusions:**

Our data indicate that appropriate EAT is associated with reduced incidence of recurrence and lower long-term mortality following bacteraemia.

**Electronic supplementary material:**

The online version of this article (doi:10.1186/s12879-017-2233-z) contains supplementary material, which is available to authorized users.

## Background

The mortality from bacteraemia remains high and bacteraemia is associated with serious patient morbidity [1–4]. Antibiotic therapy is imperative in the treatment of bacteraemia and treatment cannot await isolation and identification of the microorganisms involved and their antibiogram. Antibiotics are therefore given on an empirical basis and early initiation of appropriate empirical antibiotic treatment (EAT) has been associated with markedly improved survival in patients with septic shock [5]. Nevertheless, existing studies have provided conflicting evidence of the association between appropriate EAT and short-term mortality for bacteraemic patients [6–20]. It is likely that much of the controversy stems from heterogeneity and inadequacy in study designs, which make the comparisons of EAT and bacteraemia outcomes difficult [21, 22]. Moreover, there are little data on the association between EAT and long-term mortality following bacteraemia [23–25].

Another important adverse outcome of bacteraemia is a recurrent bacteraemic episode. Few studies have investigated recurrence of bacteraemia and even more seldom with the inclusion of all aetiologic agents [26–29]. Recent population-based studies have demonstrated that roughly one tenth of patients who survive their first bacteraemic episode experience a recurrent episode within the following year. This subsequent episode has been identified as an important predictor of mortality [26, 28, 30]. Only one prior study has investigated the association between EAT and recurrence, finding that inappropriate EAT was a predictor of recurrence [26].

The present population-based cohort study of patients with a first episode of bacteraemia aimed to examine (i) the incidence of recurrence by EAT status, and (ii) the 2–30-day and 31–365-day mortality by EAT status.

## Methods

### Setting

This population-based cohort study was conducted in 2007–2008 in three areas: the Copenhagen City area (population ~625.000, served by five public hospitals), the Copenhagen County area (population ~600.000, served by three public hospitals) and the North Denmark Region (population ~575,000, served by eight public hospitals) [31]. In total, the population base comprised approximately 1/3 of the Danish population.

Unlimited access to health services free of charge is provided to all Danish residents through a tax-funded health care program. Only few patients, e.g. in need of solid organ transplantation, were referred to hospitals outside the area in which they resided. All Danish residents have a unique civil registration number, which is used for all health-care contacts, permitting unambiguous linkage between registries [32].

### Data source

Data were obtained from the Danish Collaborative Bacteremia Network (DACOBAN), which includes the Departments of Clinical Microbiology (DCMs) in the North Denmark Region (DCM Aalborg) and the greater Copenhagen area (DCM Hvidovre and DCM Herlev) and cover the years 2000–2011 [31]. These DCMs use the same laboratory information system (ADBakt, Autonik, Sweden) and the same electronic platform is used at all three sites for real-time recording of key information on bacteraemia patients. This has allowed a uniform prospective registration of bacteraemias by physicians and a common database has been developed through linkage of data sets. The database includes the species, type, and susceptibility pattern of the bacterial and fungal isolates, origin of infection, suspected focus of infection (only for 2007–2008), and antibiotic treatment (at the time of the first notification (=EAT) and the second notification, only for 2007–2008), besides the patients’ age, gender, and day of admission. The data were linked to the Danish National Patient Registry [33] and the Danish Civil Registration System [32]. These registries hold information on the dates of admission and discharge and up to 20 physician-given discharge diagnoses, classified according to the Danish version of the International Classification of Diseases (ICD-10), and daily updated records on the vital status of all Danish residents, including date of emigration or death.

### Assessment of bacteraemia

**Fig. 1:**
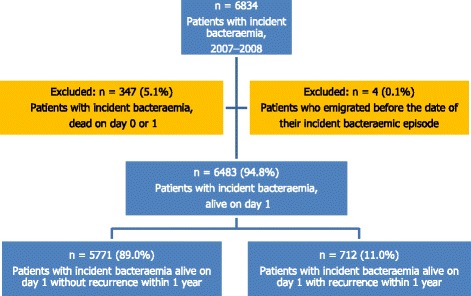
Derivation of the study population

Figure 1 describes the derivation of the study cohort. The study comprised all patients with clinically and microbiologically verified bacteraemia. To be eligible, patients had to have an incident episode of bacteraemia, i.e. no prior bacteraemic episode during the preceding 365 days. Follow-up of recurrence and vital status was conducted through 2009. Patients dying on days 0 and 1 were excluded for several reasons: bacteraemia has a time-dependent progression that reflects the dynamic interplay of the infectious agent, the host’s immune responses, and therapeutic interventions, including EAT. The critical time for antibiotics to mitigate the lethal effects of infection is generally not known, whereas other interventions including goal directed therapy have been shown to be effective within hours [34–36].

An episode of bacteraemia was defined as all clinically important blood culture isolates within the initial two days of the drawing of the sentinel blood culture (days 0 and 1) and any re-isolation of the same species within 30 days. Polymicrobial bacteraemia was defined as an episode with more than one clinically important blood culture isolate detected within the initial two days. Recurrent episodes of bacteraemia were determined by either blood culture isolate(s) of a different species obtained more than two days after the incident episode or by blood culture of the same species more than 30 days after the incident episode [26, 37, 38]. In most cases, the arbitrary 30-day limit allows for time to resolution of the infection or immediate failure of therapy [39–41].

The infection was defined as community-acquired or nosocomial according to the CDC criteria of 1988 [42]. In addition, healthcare-associated bacteraemia was defined as an episode of bacteraemia in patients with hospital contact within 30 days prior to admission (regular visits, e.g. for haemodialysis or chemotherapy, or a hospital stay) in accordance with the definitions of Friedman et al. [43] except that we had no data on home nursing or residency in nursing homes.

Data were assessed for common skin contaminants (*Bacillus* spp., *Corynebacterium* spp. *Propionibacterium* spp., *Micrococcus* spp., and coagulase-negative staphylococci). Isolation of these organisms from at least two different sets of blood draws within a 5-day period was required to fulfil the criteria for bacteraemia [44].

### Assessment of EAT

We defined EAT as the antibiotic treatment given at the 1st notification of a positive blood culture. It was recorded as appropriate if given intravenously (except fluoroquinolones, metronidazole, and fluconazole) and if all the blood isolates were susceptible to one or more of the antibiotics given. Inappropriate EAT covered recorded EAT that did not fulfil these criteria whereas unknown EAT comprised unrecorded EAT.

### Assessment of comorbidity

Charlson comorbidity index scores were calculated using discharge data from the Danish National Patient Registry as previously described [26, 45]. Three levels of the index were defined: 0 (low), corresponding to no recorded underlying diseases implemented in the index, 1–2 (medium), and >2 (high).

### Microbial identification and susceptibility testing

Blood cultures were processed using either the BacT/Alert™ system (bioMérieux, Marcy l´Etoile, France) (DCM Aalborg and DCM Hvidovre) or the BACTEC 9240™ system (Becton Dickinson, Sparks, MD, USA) (DCM Herlev). The standard method for susceptibility testing of non-fastidious bacteria was disk diffusion (NeoSensitabs, Rosco, Taastrup, Denmark (DCM Aalborg) or Oxoid, Basingstoke, UK) on Mueller-Hinton agar (DCM Aalborg) or Iso-Sensitest (ISA) medium (DCM Herlev and DCM Hvidovre). Minimum inhibitory concentration (MIC) break-points were as recommended by The Swedish Reference Group for Antibiotics with the exception that the wild-type population of *Escherichia coli* was categorized as susceptible to ampicillin at DCMs Herlev and Aalborg in accordance with national practice [46]. Susceptibility testing was standardized according to The Swedish Reference Group for Antibiotics (now NordicAST). All three laboratories use Neqas external quality control specimens.

### Statistical analyses

We considered three different study outcomes: recurrence, 2–30 day mortality, and 31–365 day mortality. The start of follow-up was defined as the date of the sentinel blood culture.

The data were first analysed using contingency tables. Covariates were categorized as follows: age (0–15, 16–64, 65–79, 80+ years), gender, Charlson comorbidity index scores (0, 1–2, >2), origin of infection (community-acquired, nosocomial, healthcare-associated, unknown), speciality (medicine, surgery, intensive-care unit, paediatrics, unknown), group of microorganisms (monomicrobial Gram-positive, monomicrobial Gram-negative, monomicrobial anaerobic, fungal, polymicrobial), and focus of infection (urogenital, respiratory, abdominal, miscellaneous, unknown).

Cumulative incidence curves of recurrence by EAT status were computed, treating death as a competing risk. The cause-specific hazard for recurrence (2–365 days) was modelled by a Cox proportional hazards approach, treating death or end of follow-up period as censored. Hazard ratios (HR) for inappropriate EAT and unknown EAT were computed with 95% confidence intervals (CIs) using appropriate EAT as reference group. The following covariates were controlled for in the adjusted analyses: age, gender, Charlson comorbidity index score, origin of infection, speciality, and group of microorganism. The focus of infection was excluded due to correlation to microorganism and because of missing values.

To illustrate mortality over time, we computed crude survival curves (2-365 days mortality) stratified by EAT. For 2-30 and 31-365 day mortality we used logistic regression analysis to compute odds ratios (ORs) with 95% CIs, using appropriate EAT as reference group, while controlling for the same covariates as listed in the Cox regression analysis.

We further reiterated the regression analyses for patients with monotherapy and combination therapy.

Statistical analyses were performed using Stata® SE (StataCorp, College Station, TX, USA).

## Results

### Patient characteristics at the incident episode

We identified 6483 patients with an incident episode of bacteraemia (Table 1). The median age was 71 years (interquartile range, 59–82 years) and 3431 (53%) were males. Appropriate EAT was given to 3778 (58%) patients, 1290 (20%) received inappropriate EAT, while the EAT status was unknown in 1415 (22%) patients. Compared with appropriate EAT, inappropriate EAT was more frequent in males, patients with nosocomial bacteraemia, polymicrobial bacteraemia, and unknown focus of infection. The proportion of patients with missing data on EAT was particularly high among patients with an unknown focus of infection (49%).

**Table 1:** Baseline characteristics of the study cohort

Variable	Categories	Empirical Antibiotic Therapy		
		Appropriate (*n* = 3778)	Inappropriate (*n* = 1290)	Unknown (*n* = 1415)
Age, years	0–15	103 (3)^a^	25 (2)	62 (4)
	16–64	1294 (34)	395 (31)	504 (36)
	65–79	1279 (34)	476 (37)	457 (32)
	80+	1102 (29)	394 (31)	392 (28)
Gender	Female	1883 (50)	539 (42)	630 (45)
	Male	1895 (50)	751 (58)	785 (55)
Charlson comorbidity index score	0	1031 (27)	305 (24)	426 (30)
	1–2	1421 (38)	476 (37)	497 (35)
	>2	1326 (35)	509 (39)	492 (35)
Origin of infection	Community acquired	2560 (68)	681 (53)	680 (48)
	Nosocomial	674 (18)	435 (34)	285 (20)
	Healthcare associated	506 (13)	150 (12)	135 (10)
	Unknown	38 (1)	24 (2)	315 (22)
Speciality	Medicine	2657 (70)	822 (64)	910 (64)
	Surgery	887 (23)	352 (27)	373 (26)
	Paediatrics	92 (2)	24 (2)	52 (4)
	Intensive-care unit	133 (4)	83 (6)	72 (5)
	Unknown	9 (0)	9 (1)	8 (1)
Group of microorganisms	Monomicrobial Gram-negative	1444 (38)	398 (31)	605 (43)
	Monomicrobial Gram-positive	2037 (54)	529 (41)	557 (39)
	Monomicrobial anaerobic	104 (3)	54 (4)	97 (7)
	Fungi	34 (1)	98 (8)	45 (3)
	Polymicrobial	159 (4)	211 (16)	111 (8)
Focus of infection	Urogenital	1287 (34)	405 (31)	224 (16)
	Respiratory	499 (13)	75 (6)	74 (5)
	Abdominal	508 (13)	204 (16)	199 (14)
	Miscellaneous	564 (15)	145 (11)	219 (15)
	Unknown	920 (24)	461 (36)	699 (49)

^a^Number (%)

Monotherapy was more frequent among patients receiving inappropriate, compared to appropriate, EAT (Table 2).

**Table 2:** Empirical antibiotic therapy in patients with a known status of empirical antibiotic therapy

Variable	Empirical antibiotic therapy	
	Appropriate (*n* = 3778)	Inappropriate (*n* = 1290)
Mono versus combination therapy		
Monotherapy	1830 (48)^a^	822 (64)
Two antibiotics in combination	1271 (34)	339 (26)
Three or more antibiotics in combination	677 (18)	129 (10)
Beta-lactam antibiotics		
Penicillin	601 (16)	279 (22)
Ampicillin	263 (7)	104 (8)
Dicloxacillin	49 (1)	30 (2)
Piperacillin/tazobactam	225 (6)	69 (5)
Mecillinam	91 (2)	106 (8)
Cefuroxime	2168 (57)	485 (38)
Third generation cephalosporin	152 (4)	21 (2)
Meropenem	96 (3)	48 (4)
No beta-lactam	133 (4)	148 (11)
Other antibiotics		
Gentamicin	900 (24)	101 (8)
Ciprofloxacin	538 (14)	176 (14)
Gentamicin & Ciprofloxacin	13 (<1)	3 (<1)
Neither	2327 (62)	1010 (78)
Metronidazole	1008 (27)	275 (21)
Antifungal agent	105 (3)	49 (4)

^a^ Number (%)

### Recurrence of bacteraemia

Within one year of the incident episode, 712 (11%) patients experienced a recurrent episode. 356 (50%) of these patients died within one year of the incident episode (compared to 2012 (35%) patients who died within one year after the incident episode without experiencing a recurrence). Among patients experiencing a recurrence, 442 (62%) were male and the median time between the two episodes of bacteraemia was 58 days (interquartile range, 21–145 days). Most recurrent episodes were community-acquired (47%), 26% were nosocomial, 23% were healthcare-associated and for 5% the acquisition was unknown. The most common group of microorganisms in recurrent episodes were monomicrobial Gram-negatives (47%), followed by monomicrobial Gram-positives (36%), polymicrobial (10%), fungi (4%), and monomicrobial anaerobes (3%).

**Fig. 2:**
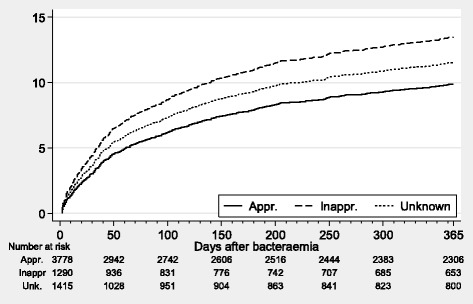
Cumulative incidence of recurrent bacteraemia following the incident bacteraemic episode, treating death as a competing risk

The incidence proportion of recurrent bacteraemia one year after the incident episode was 9.9% in patients receiving appropriate EAT, 13.6% in patients receiving inappropriate EAT, and 11.4% in patients with unknown EAT status (Fig. 2). When compared with patients receiving appropriate EAT the adjusted cause-specific HRs of 2–365 day recurrence was 1.25 (95% CI = 1.03–1.52) in patients who received inappropriate EAT and 1.31 (95% CI = 1.08–1.60) in patients with unknown EAT status (Table 3).

**Table 3:** Risk of recurrence, 2–365 days after the incident episode of bacteraemia

Empirical antibiotic therapy	Incident episode *n* (% of total)	Recurrent episode, day 2–365		
		*n* (%)	Cause-specific Hazard Ratio (95% CI)	
			Crude	Adjusted^a^
Appropriate	3778 (58)	375 (9.9)	1.0 (reference)	1.0 (reference)
Inappropriate	1290 (20)	176 (13.6)	1.50 (1.25–1.79)	1.25 (1.03–1.52)
Unknown	1415 (22)	161 (11.4)	1.23 (1.02–1.48)	1.31 (1.08–1.60)

^a^ Adjusted for age, gender, Charlson index score, origin, speciality, and group of microorganism

### Mortality after bacteraemia

**Fig. 3:**
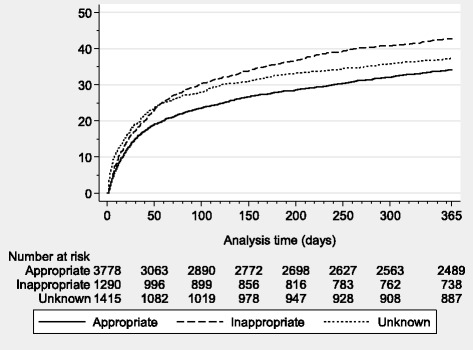
Cumulative incidence of mortality following the incident bacteraemic episode

Figure 3 presents mortality curves for patients during days 2–365 following an incident bacteraemia by EAT status. The 2–31 day mortality was 15.1% in patients who received appropriate EAT compared to 17.4% in patients who received inappropriate EAT, whereas the corresponding percentages for day 31–365 were 22.3% and 30.7%, respectively. The corresponding adjusted ORs (95% CI) were 0.85 (0.70–1.02) for 2–30 days and 1.35 (1.13–1.60) for 31–365 days (Table 4).

**Table 4:** Mortality risks, 2–30 and 31–365 days after an incident episode of bacteraemia

Empirical antibiotic therapy	Incident episode *n* (% of total)	2–30 day mortality			31–365 day mortality^a^		
		*n* (%)	Odds Ratio (95% CI)		*n* (%)	Odds Ratio (95% CI)	
			Crude	Adjusted^b^		Crude	Adjusted^b^
Appropriate	3778 (58)	571 (15)	1.0 (reference)	1.0 (reference)	716 (22)	1.0 (reference)	1.0 (reference)
Inappropriate	1290 (20)	224 (17)	1.18 (1.00–1.40)	0.85 (0.70–1.02)	327 (31)	1.54 (1.32–1.80)	1.35 (1.13–1.60)
Unknown	1415 (22)	271 (19)	1.33 (1.13–1.56)	1.26 (1.05–1.50)	259 (23)	1.02 (0.87–1.20)	1.03 (0.85–1.24)

^a^ For patients alive on day 30 (four patients who migrated between day 31 and 365 excluded)

^b^ Adjusted for age, gender, Charlson index score, origin, speciality, and group of microorganism

### Regression analyses in patients with monotherapy and combination therapy

None of the ORs or HRs deviated materially from the ORs/HRs found in the whole study cohort (Additional file 1: Table S1).

## Discussion

This population-based study demonstrates that inappropriate EAT is associated with an increased risk of bacteraemia recurrence and of long-term mortality. We found that 11% of the bacteraemic patients experienced a recurrent episode and the 1-year recurrence incidence proportion was 1.4-fold higher for patients receiving inappropriate EAT than for patients receiving appropriate EAT.

Our findings are consistent with prior studies that have found recurrence rates around 10% in both non-selected and selected populations [26–30, 47, 48]. Our finding of inappropriate EAT as an independent predictor of recurrent bacteraemia is in accordance with another Danish bacteraemia cohort for which we found an adjusted OR of 1.29 (95% CI = 1.10–1.52) [26].

Inappropriate EAT was not associated with short-term (2–30 days) mortality, an outcome measure that has been studied intensively. Similar to our findings, several studies have reported that inappropriate EAT was not associated with short-term mortality with the cautions that most of these studies included relatively few patients and investigated either individual microorganisms or groups of microorganisms or only reported in-hospital mortality [6, 11, 12, 20, 49–52]. Nonetheless, the majority of studies focusing on short-term mortality have shown that inappropriate EAT is associated with all-cause mortality as summarised by two major reviews [21, 22]. Both reviews pointed out that heterogeneity in study design, study populations, time to outcome measures and methodological pitfalls (e.g. the definition of inappropriate EAT and the distinction between empirical and definitive treatment) contributes to the conflicting results.

In contrast, inappropriate EAT was a prognostic factor for long-term mortality (31–365 days).

Our study’s 1-year mortality of 39.7% (including the 347 dying on day 0 or 1, cf. Fig. 1) is in accordance with the few other population-based bacteremia studies that have assessed this [25, 53]. Long-term mortality of bacteraemia is related to age and underlying disorders while infection presumably plays a less direct role, although the nature and severity of the initial infection as well as direct and indirect complications arising from the acute disease (e.g. organ dysfunction) may lead to considerable mortality months or years later [25, 54]. Appropriate EAT may reduce the harmful inflammatory response leading to organ dysfunction by clearing the pathogen and thereby influencing long-term mortality. The positive association between inappropriate EAT and long-term mortality has previously been demonstrated for *Staphylocuccus aureus* bacteraemia in a hospital-based study [23, 24], but to the best of our knowledge only in one study with a non-selected patient population comprising all common bacteraemia microorganisms [25].

The strengths of our study include the population-based design, a large cohort, the recording of the patients’ clinical data by physicians during the course of disease, and virtually complete follow-up. Still, there are limitations that require consideration. Firstly, our retrospective study data were obtained through existing databases in which the clinical data were inadequate. In particular, 22% of the patients lacked information on EAT. We categorized these patients separately which may have introduced bias. Due to limited clinical data, we were not able to control for baseline severity of illness, which is one important recommendation in designing outcome studies of bacteraemia according to EAT [21, 22]. However, we adjusted for speciality as a surrogate marker for the acute disease. Secondly, selected data from the DACOBAN research database were from 2007 to 2008 though newer data exist. This was due to physicians’ meticulous and prospective registration of clinical information related to the focus of infection and antibiotic treatment during these two years. This partly compensated for the inadequacy of clinical data. We do not believe that data of newer antibiotic treatment regimes in recent years would materially alter the results, which conform to studies from other settings [12, 50]. Thirdly, the patient populations from the three DCMs were heterogeneous in several respects. Fourthly, the clinical data were recorded by many different registrars and consultants leaving room for both missing data and variations reflecting individual judgement which may lead to information bias. Also, ampicillin MIC break-points were defined differently at DCM Hvidovre, which may have introduced misclassification bias. However, this error potentially affected less than 10 patients as ampicillin was rarely given as monotherapy. Fifthly, we cannot rule out confounding by indication as the efficacy of the different empirical antibiotic regimens may not have been homogeneous. Confounding by indication is an inevitable problem in observational studies that involve drugs and the severity of illness can lead to a broader coverage of EAT, especially in acute infectious disorders. We distinguished between appropriate and inappropriate EAT, but did not stratify for which of the antibiotic(s) that provided the appropriate EAT, e.g. a betalactam or an aminoglucoside, nor dosage regimes and the actual timing of EAT, which is important as shown previously [5, 7].

## Conclusions

This study showed that inappropriate EAT was a predictor of recurrent bacteraemia and increased the long-term mortality following bacteraemia, whereas it had no impact on the short-term mortality. These findings have clinical importance and highlight the importance of vigilance in the identification and antibiotic treatment of bacteraemia. Identification of patients and characteristics associated with inappropriate EAT may contribute to empirical prescribing guidelines and thereby improve EAT.

## Additional file

Additional file 1: Table S1. Table heading: Mortality and recurrence risks for inappropriate empirical antibiotic therapy (EAT) patients (appropriate EAT patients is the reference group). (DOCX 14 kb)

## Abbreviations

CI: Confidence interval
DCM: Departments of Clinical Microbiology
EAT: Empirical antibiotic treatment
HR: Hazard ratio
ICD: International Classification of Diseases
MIC: Minimum inhibitory concentration
OR: Odds ratio
