# Supplementary material for: Impact of appropriate empirical antibiotic treatment on recurrence and mortality in patients with bacteraemia: a population-based cohort study
Source: BMC Infect Dis. 2017 Feb 6;17:122. doi: 10.1186/s12879-017-2233-z (PMC5294810; doi:10.1186/s12879-017-2233-z)
Supplement: Additional file 1: Table S1. — Table heading: Mortality and recurrence risks for inappropriate empirical antibiotic therapy (EAT) patients (appropriate EAT patients is the reference group). (DOCX 14 kb) [file 12879_2017_2233_MOESM1_ESM.docx]

| **Patients** | **2-30 d mortality** | | **31-365 d mortality** | | **2-365 day recurrence** | |
| --- | --- | --- | --- | --- | --- | --- |
|  | **Odds ratio (95% CI)** | | **Odds ratio (95% CI)** | | **Hazard ratio (95% CI)** | |
|  | **Crude** | **Adjusted*** | **Crude** | **Adjusted** | **Crude** | **Adjusted** |
| All | 1.18 (1.00-1.40) | 0.85 (0.70-1.02) | 1.54 (1.32-1.80) | 1.35 (1.13-1.60) | 1.50 (1.25-1.79) | 1.25 (1.03-1.52) |
| Monotherapy | 1.21 (0.96-1.53) | 0.93 (0.71-1.22) | 1.38 (1.13-1.69) | 1.17 (0.93-1.48) | 1.67 (1.30-2.15) | 1.48 (1.12-1.96) |
| Combination therapy | 1.31 (1.02-1.68) | 0.91 (0.68-1.22) | 1.85 (1.44-2.36) | 1.65 (1.24-2.19) | 1.53 (1.17-1.99) | 1.19 (0.88-1.60) |

* Adjusted for age, gender, Charlson index score, origin, speciality, and group of microorganism
